# Medical specialty preferences in early medical school training in Canada

**DOI:** 10.5116/ijme.59f4.3c15

**Published:** 2017-11-14

**Authors:** Anthony Vo, Laurie McLean, Matthew D.F. McInnes

**Affiliations:** 1 Department of Diagnostic Radiology, University of Alberta, Canada; 2 Department of Otolaryngology-Head and Neck Surgery, University of Ottawa, Canada; 3 Department of Diagnostic Radiology, University of Ottawa, Canada

**Keywords:** Medical education, undergraduate, medical students, career, counseling

## Abstract

**Objectives:**

To understand what medical students consider when choosing their specialty, prior to significant clinical exposure to develop strategies to provide adequate career counseling.

**Methods:**

A cross-sectional study was performed by distributing optional questionnaires to 165 first-year medical students at the University of Ottawa in their first month of training with a sample yield of 54.5% (n=90).  Descriptive statistics, analysis of variance, Spearman's rank correlation, Cronbach's alpha coefficient, Kaiser-Meyer-Olkin Measure, and exploratory factor analyses were used to analyze the anonymized results.

**Results:**

“Job satisfaction”, “lifestyle following training” and, “impact on the patient” were the three highest rated considerations when choosing a specialty.  Fifty-two and seventeen percent (n=24) and 57.89% (n=22) of males and females ranked non-surgical specialties as their top choice. Student confidence in their specialty preferences was moderate, meaning their preference could likely change (mean=2.40/5.00, SD=1.23). ANOVA showed no significant differences between confidence and population size (F_(2,86)_=0.290, p=0.75) or marital status (F_(2,85)_=0.354, p=0.70) in both genders combined. Five underlying factors that explained 44.32% of the total variance were identified. Five themes were identified to enhance career counseling.

**Conclusions:**

Medical students in their first month of training have already considered their specialty preferences, despite limited exposure. However, students are not fixed in their specialty preference. Our findings further support previous results but expand what students consider when choosing their specialty early in their training. Medical educators and administrators who recognize and understand the importance of these considerations may further enhance career counseling and medical education curricula.

## Introduction

In medical schools, students are exposed to Family Medicine and a vast number of specialties during their training. Eventual specialty choices available are broad and varied-each encompassing a particular patient demographics, skill set, disease spectrum, income, lifestyle, and a multitude of other factors. The process of choosing a medical specialty is complex. The goal is for a student to choose a specialty that best meets his or her needs and desires while at the same time meeting society's needs.^1^ However, the process, including the starting point in Undergraduate Medical Education (UGME), that a student uses to determine his/her eventual medical specialty choice is not well understood.

Similarly, the perceived career counseling needs of students to help them with their specialty choice has not been well studied.

There are several Canadian studies that have explored what students consider when choosing their specialty; however these have been specialty specific, looking at differentiated students who have chosen their specialty, which may make it difficult for medical educators and administrators to apply the study findings more broadly especially when counseling a student who has not yet made a specialty choice early in UGME.^2^^-^^20^ For example, Scott et al. found that students choosing surgery were less influenced by considerations such as medical lifestyle, the varied scope of practice, and social orientation.^16^ Alternatively, those who chose Family Medicine were influenced by future practice, nonpractice life considerations, and postgraduate training.^8^ Although students graded these considerations using categorical scales, such as a Likert scale, these methods may have obscured the true stratification and importance of these on specialty choice. For example, Scott and colleagues used a 5-point Likert scale to grade what students consider when choosing a specialty, which may have made it difficult to determine the precise influence of each of these given that each point was a 20% increment.^14^ Furthermore, the importance of what students consider may change with time, location, culture, technology, healthcare structure, government policy, age, workforce etc.^2^^,^^3^^,^^21^^-^^24^For example, in a 2013 Spanish study, the authors identified that following an economic crisis, What was considered important, such as prestige and quality of life, were replaced with income and job security.^25^ Similarly, workforce and job security may matter in Canada, where certain specialties are overrepresented, while others are underrepresented.^26^^,^^27^

These complex and ever-changing issues are challenging to navigate. Objective data that can enhance career counseling is imperative. If equipped with this broad knowledge, medical educators and administrators may further improve educational strategies, curricula and career counseling services to best meet the needs of medical students. Therefore, the objective of this study is to understand what medical students consider when choosing their specialty, prior to significant clinical exposure to develop strategies to provide adequate career counseling. We hypothesized that the considerations students use in the current healthcare system are different from prior findings in differentiated students who have chosen their specialty.

## Methods

### Study design and participants

Ethical approval was obtained from the Ottawa Health Sciences Research Ethics Board. Using convenience sampling, the participants of this study included first-year medical students at the University of Ottawa, in both the Anglophone (n=117) and Francophone (n=48) streams. A certified translator translated all documents distributed to Francophone students. The study was conducted within the first month of medical school for three reasons. First, it would serve as baseline data which could then be used in comparison to data from later UGME years to understand best if and how students transition in specialty choice. Second, it would provide the opportunity to understand the specialty preconceptions held by UGME students prior to significant medical exposure. Third, it allows medical educators and administrators to understand how fixed students are in their specialty choice early in their training.

Of the 165 eligible first-year medical students at the University of Ottawa, 90 students (64 Anglophone and 26 Francophone) returned the questionnaire. This resulted in a sample yield of 54.70% and 54.17% in the Anglophone and Francophone streams respectively, with an overall yield of 54.5%. All questionnaires met the inclusion criteria. Of the respondents, 52 participants were males, and 38 were females, with a mean age of 22.49 (range= 20.00-32.00, SD= 1.95). Ninety-four and forty percent of students were 20-25 years of age, 3.33% were 26-30 years of age, and 1.11% were 31 years of age or over. With regards to the hometown population size, 71.10%, 15.60% and 13.30% of students were from large (population 100,000+), medium (population 30,000-99,999) and small towns (population 1-29,999), respectively. These results were similar to the class demographics.

### Data collection methods

A literature review was performed to identify previous considerations that were used in choosing a specialty. These were then incorporated into a questionnaire, which included both quantitative and qualitative components.

The final questionnaire consisted of 38 questions (7 questions related to socio-demographic and background, 27 questions related to possible considerations and their importance in choosing a specialty choice, and four miscellaneous questions which included career counseling)(see Appendix A). Socio-demographic and background information (gender, marital status, age, hometown population size, and confidence in specialty choice) were used to perform subgroup analyses.

Participants were then asked to rank their top three specialty choices these included both Family Medicine and specialties that were officially recognized by the Royal College of Physicians and Surgeons of Canada.^28^ If the student did not have a preference, they were asked to select unsure. Choices were then grouped into 3 core groups based on the specialty's general scope of practice: non-surgical specialties (Pediatrics, Family Medicine, Internal Medicine, Emergency Medicine, Dermatology, Neurology, Physical Medicine and Rehabilitation, Psychiatry, and Radiation Oncology), surgical specialties (Ophthalmology, Otolaryngology, Obstetrics and Gynecology, General Surgery, Orthopedic Surgery, Neurosurgery, Cardiac Surgery, Plastic Surgery, Urology and Vascular Surgery) and auxiliary specialties (Anesthesiology, Diagnostic Radiology and Public Health and Preventive Medicine, Medical Genetics, Medical Microbiology, Transfusion Medicine, Pathology, Medical Biochemistry and Nuclear Medicine).

Students were asked to rate how confident they were of their specialty choices, with values ranging from 1 (it will most likely change - I have not begun to explore the various career options yet) to 5 (it will not change-I am sure of the specialty I want to enter at this time in my training). Questions related to the importance of the listed considerations were rated on a continuous scale from 0 (not important or doesn't affect specialty choice) to 100 (critical). This scale was used instead of a categorical scale, such as a Likert point scale, to stratify their importance better.Students were then asked to rate their current knowledge of career counseling services available to them using a Likert scale ranging from 1 ("I do not know of any resources available to me") to 5 ("I am aware of sufficient resources available to me").

### Procedure

Students were voluntarily recruited. The purpose of the study, the benefits and risks were explained prior to voluntary consent. Hardcopies of the consent form and the questionnaire were distributed to the students. To encourage thoughtful responses, students were asked to return these documents at a later date to allow time to reflect. The questionnaire was anonymized prior to statistical analyses. These tasks were not performed by the authors.

### Data analysis

Each submission was screened to ensure completeness to be eligible for inclusion. Questionnaires with at least 80% of the questions correctly completed were included. Analyses of the data were then performed using SPSS software package (version 20; SPSS, Chicago, Illinois).

Results were analyzed as a single group and as sub-groups, stratified by the results from the socio-demographic and background questions. Analyses for differences included analysis of variance (ANOVA), to analyze for the significance of the means between different groups. Spearman's rank correlation was used to model the linear relationship between two variables. Interpretation of the Spearman's rank correlation coefficient value was as followed:±0.00-0.25= weak, ±0.25-0.50=fair, ±0.50-0.75-moderate, >±0.75=very strong.^29^

Exploratory factor analysis was performed to group the considerations by determining possible underlying factors statistically. A Kaiser-Meyer-Olkin Measure (KMO) of sampling adequacy value of at least 0.50 was required. Interpretation of the value was as followed: 0.50-0.70=mediocre, 0.70-0.80=good, 0.80-0.90=great, >0.90=superb.^30^ The listed considerations needed to demonstrate a minimum factor loading of 0.5 to show evidence of a strong relationship between the considerations and each new factor. At least two considerations loaded onto a factor were required to provide meaningful interpretation.^31^^-^^32^ Considerations that clustered into new factors that demonstrated more variance (Eigenvalue greater than 1) were retained. Cronbach's alpha coefficients were calculated to estimate the internal consistency of the considerations. The general guideline for the interpretation of the value are as followed: <0.5=unacceptable, 0.5-0.6=poor, 0.6-0.7=questionable, 0.7-0.8=acceptable, 0.8-0.9=good, >0.9=excellent.^33^ Inter-item correlation was used to maximize internal consistency of the considerations in each factor in order to better interpret the results. The authors interpreted the considerations within each factor to determine and describe an appropriate underlying factor that could explain the considerations it encompassed. Descriptive statistical analyses were performed to determine the mean, standard deviation (SD), and range. Qualitative responses were aggregated together by similarity to identify themes. Given that the purpose of this study was exploratory, a p-value less than 0.05 was considered statistically significant.

## Results

Pertaining to the students' top three specialty choices, 52.17% (n=24), 63.00% (n=29), 55.32% (n=26) of males ranked non-surgical specialties as their first, second and third choice respectively. In females, 57.89% (n=22), 68.42% (n=26), 57.89% (n=22) ranked non-surgical specialties as their first, second and third choice respectively. The top three non-surgical specialties ranked first by both genders combined were Family Medicine, Pediatrics, and Emergency Medicine. Auxiliary specialties remained the least popular choice for both genders.

The students' confidence in their ranking was quantified. Male and female students had a mean score of 2.41 (SD=1.34) and 2.39 (SD=1.08) out of 5.00 respectively, without a significant difference between the two genders (F_(__1,87)_=0.004, p-value=0.95). With both genders combined, the mean value was 2.40 (SD=1.23). The students' confidence was then sub-analyzed by their hometown population size, marital status, and age. ANOVA showed no significant differences in the mean values between the three population sizes for males (F_(__2,48)_=0.022, p=0.98), females (F_(2,35)_=0.432, p=0.65), and both genders combined (F_(2,86)_=0.290, p=0.75). Moreover, there were no differences between confidence and the students' marital status in males (F_(__2,48)_=0.982, p=0.38), females (F_(2,34)_=0.103, p=0.90), and both genders combined (F_(2,85)_=0.354, p=0.70). Spearman's rank correlation did not reveal significant linear relationships when confidence was correlated with population size, marital status and age in males, females and both genders combined.

When students had to choose the single most important consideration, "job satisfaction" (n=21), "lifestyle following training" (n=18), and "future job market" (n=8) tied with "personal fit into the specialty" (n=8) were ranked as the highest. When students were asked to rate the importance of each listed consideration from 0 to 100, there were no significant differences between males and females for all the considerations. For both genders combined, "job satisfaction" (mean=87.81 and SD=13.33), "lifestyle following training" (mean=83.31 and SD=16.18), and "impact on the patient" (mean=82.2 and SD=14.21) were the three highest rated considerations.

The KMO of sampling adequacy value was 0.71, which suggested adequate sampling and that the listed considerations were suitable for exploratory factor analysis. Five underlying factors were identified which explained 44.32% of the variance. The five factors were "perception of the specialty" (consisting of 3 considerations, Alpha=0.70, 16.08% of the variance explained), "specialty's practice" (consisting of 3 considerations, Alpha=0.72, 9.53% of the variance explained), "job prospects" (consisting of 3 considerations," Alpha=0.42, 7.04% of the variance explained), "knowledge of the specialty" (consisting of 4 considerations, Alpha=0.77, 5.99% of the variance explained), and "social orientation" (consisting of 2 considerations, Alpha=0.50, 5.68% of the variance explained). Given that some factors were loaded with a small number of considerations, all five factors were considered to have valid internal consistency given that a lower reliability was deemed to be acceptable in these instances.

Students were asked to rate their current knowledge of career counseling services available to them. The mean score of both genders combined was 2.41 (SD=1.00). Five themes were identified following qualitative analyses.

The first theme was Enhanced Communications. Students were not aware of resources available to them to help choose a specialty. They preferred clear and transparent communication regarding resources available. They wanted the communications to be honest, practical and realistic so that they could apply them. Some comments focused on the residency matching process rather than the specialty itself. For example, a male student wrote, "more 'realistic' advice on how to get into the specialty we want ..." (study id 95358714).

The second theme was Career Resources. Students emphasized having career-related resources that were practical to them. These included documents, pamphlets, and databases that were specialty specific. Students emphasized that resources should include how to decide a specialty, relevant information about job prospects and suggestions as to how to decide a specialty. Students would like contact information to connect with residents and physicians in these specialties. For example, a female student commented, "give an idea of what specialty could interest us; have a booklet with relevant information about job prospects; match us with an appropriate mentor to guide us if we decide on the specialty" (study id 95347748).

The third theme was Presentations and Seminars. Having frequent, honest and personal presentations from members of different specialties were important. Students wanted to learn from the personal experience of residents and staff, along with how they chose their specialty. For instance, a male student wrote, "incorporate guest speakers from different specialties into the curriculum..." (study id 95444370), and a female student wrote, "speakers/residents/students discuss their experiences and how they went about choosing their specialty" (study id 95343778).

The fourth theme was Opportunities for Exposure. Students wanted early opportunities to be exposed and to interact with those in the specialty of interest. These included opportunities to meet, shadow, and work. For example, a female student wrote, "opportunities to meet with members in fields of interests, to shadow them" (study id 95346040).

The fifth theme was Counseling Services. Students reported interest in services that help them align their personal characteristics with various specialties. These included frequent guidance by counselors, personality tests, reflective sessions, and workshops to identify potential specialties. For example, a male student wrote, "I would like to see mandatory counseling sessions, similar to high school guidance counseling" (study id 95284102).

## Discussion

The students' specialty preconceptions at the beginning of medical school are important to highlight because they predict the student's ultimate specialty in 45-70% of cases.^3^ In our study, the majority of students ranked non-surgical specialties as their first, second and third choice. These are suggestive of the current emphasis on specialties that provide more flexibility and control over work hours.^34^

However, the study findings also infer that regardless of the students' socio-demographic differences (hometown population size, age, and marital status), they are not confident in their specialty rankings. This suggests that medical educators and administrators may be able to equally counsel students, regardless of the students' socio-demographic differences.

Previous studies have used categorical scales, such as a Likert scale, to grade the importance of the considerations used to choose a specialty, which may have obscured the true stratification and influence of these. In our study, a continuous scale was used to clarify the influence further. Thus, overall "job satisfaction" (mean=87.81 and SD=13.33), "lifestyle following training" (mean=83.31 and SD=16.18), and "impact on the patient" (mean=82.2 and SD=14.21) were identified as the three with the highest rated mean. If categorical scales were used, these might have been difficult to differentiate.

In previous studies which explored what students considered when they have already chosen a specialty, job satisfaction, lifestyle and patient impact were similarly identified as important considerations.^6^^,^^8^ Our findings further support these but generalizes the importance of these to medical students early in their training. Furthermore, our results suggest that there were no significant differences between males and females for the listed considerations. When students were asked to choose the most important consideration, "future job market" was noted, which further exemplified the concerns students may have about the high unemployment rates faced by various specialties even before students complete their first month of medical training.^35^

Five underlying factors were identified which accounted for 44.32% of the total variance. From a medical educator and administrator point of view, including discussion about these considerations that are highly regarded among students may significantly impact career counseling in a positive manner.

There are several limitations with this study. It presented a cross-sectional sample of one class consisting of two language streams (Anglophone and Francophone) at the University of Ottawa. Greater inferences may have been drawn if multiple institutions from both languages were sampled. It is difficult to know if what students consider when choosing a specialty in Ontario are similar to other provinces. Furthermore, this study was administered to students within the first month of medical training, and, as the students suggested, it is possible and likely that the student's perspectives will change as they begin to understand further the medical profession to which they have aspired. Similarly, as they are very early in their training and moving through an identified significant transition time, it is highly possible that they are not yet aware of the current career guidance programs and curricula already in place at their university.  A future study could not only reexamine the considerations students use at various time points in medical school but also explore the most effective times in medical school for multiple components of career guidance. Understanding the timing, if any, of the transition of not only what students consider, but also their importance could again help to inform curricula and career counseling to best meet the most relevant concerns of the student through his or her training.

## Conclusions

Most medical students have already considered a specialty, despite limited exposure. However, they believe that there is a moderate likelihood that they will change their mind before deciding on specialty choice at the end of their undergraduate medical training, regardless of the sociodemographic differences. Our findings further support previous studies that evaluated differentiated students who have chosen their specialty but expand the importance of these considerations among medical students early in their training. Medical educators and administrators who recognize and understand the importance of these considerations may further enhance career counseling and medical education curricula. Addressing these considerations by using the five career counseling themes identified may be an initial step. Future research may explore which considerations are important to students at various specific time points in medical school and determining which type of career guidance is most useful for each time point.

### Acknowledgments

The authors of this study would like to thank Dr. Timothy Wood for his time, statistical expertise and assistance. The authors of this study would also like to thank the Department of Innovation in Medical Education, University of Ottawa, and the University of Ottawa Department of Radiology Research Stipend Program for funding this study.

### Conflict of Interest

The authors declare that they have no conflict of interest.

## Supplementary material

Supplementary file 1 Appendix A. Questionnaire
