# Supplementary material for: Medical specialty preferences in early medical school training in Canada
Source: Int J Med Educ. 2017 Nov 14;8:400–7. doi: 10.5116/ijme.59f4.3c15 (PMC5694695; doi:10.5116/ijme.59f4.3c15)
Supplement: Supplementary file 1 — Appendix A. Questionnaire [file ijme-8-400-S1.pdf]

Appendix A. Questionnaire

Demographics

1. Gender: (please circle one)
   a. Male b. Female

2. Marital status: (please circle one)
   a. Single b. In a serious relationship c. Married

3. Age: please write your age below
   ....

4. Medical education stream: (please circle one)
   a. English b. French

5. Home town population: (please circle one)
   a. large (population 100,000+)
   b. medium (population 30,000-99,999)
   c. small (population 1-29,999)

6. Current specialty of interest: (Please rank your top 3 choices from 1= first choice to 3= third choice)

___ Anesthesiology
___ Cardiac surgery
___ Dermatology
___ Diagnostic radiology
___ Emergency medicine
___ Family Medicine
___ General surgery
___ Internal medicine
___ Medical biochemistry
___ Medical genetics
___ Medical microbiology
___ Neurology
___ Neurosurgery
___ Nuclear medicine
___ Obstetrics and gynecology
___ Ophthalmology
___ Orthopaedic surgery
___ Otolaryngology-Head and Neck Surgery
___ Pathology
___ Pediatrics
___ Physical medicine and rehabilitation
___ Plastic surgery
___ Psychiatry
___ Public health and preventive medicine
___ Radiation oncology
___ Transfusion medicine
___ Urology
___ Vascular surgery
___ Unsure

7. How confident are your choices in Question 6? (Please circle a number from 1 to 5)

- 1=It will most likely change—I have not begun to explore the various career options yet
- 5=It will not change-I am sure of the specialty I want to enter at this time in my training

1 2 3 4 5

Factors influencing

Please write a value for each question below from 0=not important/doesn't affect to 100=critical

No.	Question Stem	Value 0-100
8	Control/predictability over work schedule (for example: on-call, commitment to work)	
9	Expected financial income	
10	Intellectual content of the specialty	
11	Scope of practice of the specialty	
12	Future job market (for example: employment opportunities, ease of employment, demand of the specialty, job security)	
13	Length of training required to practice (for example: length of residency program, fellowships)	
14	Job flexibility (for example: ease of job relocation, dependency on hospital resources [operating rooms], transferable, work location, career opportunities, opportunity for private practice)	
15	Availability of residency training placements available/ Competition for obtaining a residency position (for example: specialty competitiveness)	
16	Characteristics of the patient population (for example: pediatrics, adult, geriatrics, acute/long-term care)	

17	Lifestyle during training	
18	Lifestyle following training (for example: controllable, time for family, focus on other interests/hobbies)	
19	Influence of an attending physician's or resident's opinions and reflections (for example: advice, why they chose specialty, specialty satisfaction)	
20	Perceived working environment (for example: stressful, high-pressure, fast-paced)	
21	Patient-physician interaction	
22	Influence of a personal mentor or a teaching faculty member	
23	Influence of family members and friends	
24	Prior knowledge or skill set in the specialty	
25	Clinical or educational exposure/experience into specialty	
26	Debt and student loans that have been incurred before practicing medicine	
27	Job satisfaction	
28	Impact on the patient	
29	Treatment outcomes of patients in the specialty (for example: palliative care versus cosmetic surgery)	
30	Future opportunities to progress or sub-specialize	
31	Gender distribution in specialty (for example: low prevalence of females in surgery)	
32	Personal fit into the specialty (for example: share the same interests, personality, skills and values as those in the field)	
33	Opportunities to perform procedures/techniques	
34	Status/prestige of the specialty	

Misc Questions

35. Single most important factor from question 8-34:

(Please write question):

………..

36. Current knowledge of career counseling services available to you to help choose a medical specialty: (Please circle a number from 1 to 5)

- 1=I do not know of any resources available to me
- 5=I am aware of sufficient resources available to me

1 2 3 4 5

37. What would you like to see in terms of career counseling in medical school? (Please write any comments in the box)

38. Any other comments you might have: (Please write any comments in the box)

I would like to be contacted for the possibility of participation in future studies

Yes - University of Ottawa Email address (i.e. AVO025@uotttawa.ca): ………………………………………………….
No
